# *GDF15(MIC1)* H6D Polymorphism Does Not Influence Cardiovascular Disease in a Latin American Population with Rheumatoid Arthritis

**DOI:** 10.1155/2015/270763

**Published:** 2015-05-18

**Authors:** Jenny Amaya-Amaya, Adriana Rojas-Villarraga, Nicolas Molano-Gonzalez, Laura Montoya-Sánchez, Swapan K. Nath, Juan-Manuel Anaya

**Affiliations:** ^1^Center for Autoimmune Diseases Research (CREA), School of Medicine and Health Sciences, Universidad del Rosario, Carrera 24 No. 63C-69, Bogotá, Colombia; ^2^Arthritis and Clinical Immunology Research Program, Oklahoma Medical Research Foundation, 825 NE 13th Street, Oklahoma City, OK 73104, USA

## Abstract

*Objective*. Rheumatoid arthritis (RA) is the most common autoimmune arthropathy worldwide. The increased prevalence of cardiovascular disease (CVD) in RA is not fully explained by classic risk factors. The aim of this study was to determine the influence of rs1058587 SNP within *GDF15(MIC1)* gene on the risk of CVD in a Colombian RA population. *Methods*. This was a cross-sectional analytical study in which 310 consecutive Colombian patients with RA and 228 age- and sex-matched controls were included and assessed for variables associated with CVD. The mixed cluster methodology based on multivariate descriptive methods such as principal components analysis and multiple correspondence analyses and regression tree (CART) predictive model were performed. *Results*. Of the 310 patients, 87.4% were women and CVD was reported in 69.5%. Significant differences concerning *GDF15* polymorphism were not observed between patients and controls. Mean arterial pressure, current smoking, and some clusters were significantly associated with CVD. *Conclusion*. *GDF15* (rs1058587) does not influence the development of CVD in the population studied.

## 1. Introduction

Rheumatoid arthritis (RA) is the most common autoimmune arthropathy worldwide. It is a chronic, multifactorial, and systemic disease characterized by the presence of long-standing inflammation [1, 2]. As with most autoimmune diseases, RA predominantly affects women [2, 3], and its prevalence had been reported to be between 0.3 and 1.6% [4]. In addition to diarthrodial joints, RA can damage virtually any organ thus leading to potential extra-articular manifestations (EAMs), including cardiovascular disease (CVD) [5, 6]. CVD is the major predictor of poor prognosis and represents the main cause of death in this population [7, 8]. It accounts for 30–50% of all deaths in RA patients [9] and the prevalence of CVD in RA Latin American population has been estimated to be around 35% [8].

In the etiological and pathogenic mechanisms of CVD in RA the inflammatory activity plays an important role. Several studies assessing traditional risk factors for CVD in RA have been published [10]. Nevertheless, the increased prevalence of cardiovascular events in RA is not fully explained by these classic risk factors. Nontraditional risk factors have also been identified and categorized into three groups: genetic, RA-related, and others [7]. Recently, an association between a nonsynonymous single nucleotide polymorphism (SNP) at growth differentiation factor 15 (*GDF15*) gene (i.e., rs1058587) and CVD in RA patients was reported [11].

*GDF15,* also known as macrophage inhibitory cytokine-1 (*MIC1*), depending on the tissue that was described, is a growth and differentiation factor which belongs to the superfamily of transforming growth factor-beta (TGF-*β*). *GDF15* plays multiple roles in various pathologies such as CVD, obesity, inflammation, and cancer due to its anti-inflammatory, antiproliferative, and antitumoral properties [11–13].

Its mechanism of action is not fully elucidated but it is believed that* MIC1* activates the TGF-*β* receptors. Specifically in inflammation, it can limit the later phases of macrophage activation, inhibit the production of tumoral necrosis factor-*α* (TNF-*α*) induced by lipopolysaccharides, and also regulate the role of the proinflammatory cytokine interleukin-6 (IL-6) [10]. Additionally,* MIC1* influences metabolism of carbohydrates and lipids. For instance, patients with obesity and type 2 diabetes mellitus (T2DM) have the highest serum concentrations of this factor [14].

Previous studies have found that some* GDF15(MIC1)* polymorphisms are associated with different atherothrombotic manifestations such as stroke, transient ischemic attack, deep vein thrombosis, and pulmonary embolism.* GDF15(MIC1)* had also been associated with RA* per se, *proposing that it acts as a proinflammatory cytokine as well as a common etiologic factor for RA and CVD [11, 12]. The aim of this study was to determine the influence of rs1058587 SNP within* GDF15(MIC1)* gene on the risk of CVD in a Colombian RA population. 

## 2. Methods

### 2.1. Study Population

This was a cross-sectional analytical study in which 310 consecutive Colombian patients with RA and 228 age- and sex-matched controls were included. The sample size was not calculated. This was a nonprobability sample. The subjects were seen at the Center for Autoimmune Diseases Research (CREA) in Medellín and Bogotá, Colombia. Patients fulfilled the 1987 American College of Rheumatology (ACR) classification criteria for RA [15]. This study was undertaken between February 2008 and April 2010 and done in compliance with Act 008430/1993 by Ministry of Health of the Republic of Colombia. The institutional review board of the Universidad del Rosario approved the study design.

Each patient was evaluated by a rheumatologist. The information on patient sociodemographic and cumulative clinical and laboratory data was obtained by interview, physical examination, and chart review. Household description by questionnaire and clinical evaluation of the affected family members was done with the same methodology as above. All data were collected in an electronic and secure database.

Sociodemographic variables included age at RA onset, disease duration, educational status, socioeconomic status (SES), current occupation, smoking habits, and physical activity. Definitions of these variables are as follows. Age at onset (AOD) is the age at which patients began to suffer from pain, typical morning stiffness (more than 1 hour), and symmetrical inflammation of hand and/or foot joints. Disease duration is the difference between AOD and the date of first participation in the study. Educational level was recorded as the number of years of education and was also divided into two groups—more or less than 9 years of education based on the General Law of Education in Colombia laws [16, 17]. SES was categorized on the basis of national legislation and was divided into high status, medium status, and low status. Information on current occupational status was also requested. Familial autoimmunity (FA) was defined as the presence of any AD in first degree relatives (FDRs) of the proband [18].

Erosions were defined as having at least one unequivocal cortical bone defect evaluated by a rheumatologist [19]. EAMs were defined as the presence of at least one of the following: skin and lung nodules, skin ulcerations, episcleritis, vasculitis, neuropathy, pleural effusion, pulmonary hypertension, or embolism. CVD was categorized as positive if any of the following variables were present: hypertension, coronary artery disease (CAD), occlusive arterial disease, carotid disease, or thrombosis [20]. Regarding medical treatment, current or past use of methotrexate and other conventional disease modifying antirheumatic drugs (DMARDs) such as sulfasalazine, D-penicillamine, gold salts, and leflunomide; biological therapy (etanercept, infliximab, adalimumab, abatacept, tocilizumab, and rituximab); and azathioprine and cyclosporine were also assessed. In addition, steroid therapy (prednisolone, methylprednisolone, and deflazacort) and antimalarials (chloroquine and hydroxychloroquine) were taken into account.

Autoantibodies including rheumatoid factor (RF), anti-citrullinated protein (ACPA), and anti-nuclear antibodies (ANA) were extracted from the patient's clinical record.

### 2.2. Assessment of Traditional Risk Factors for CVD

Patients were assessed for traditional CVD risk factors including current age (≥45 and ≥55 years for men and women, resp.) [21] and history of premature CAD in FDR [22], and all individuals were asked about smoking and physical activity [23]. T2DM was defined as having a fasting plasma glucose level ≥7 mmol/L (126 mg/dL) or taking any antidiabetic agents at the time of assessment [24]. A diagnosis of dyslipidemia was given if the patient had (a) hypercholesterolemia, defined as taking lipid-lowering medication as a surrogate or having a fasting plasma total cholesterol ≥200 mg/dL, (b) HDL ≤40 mg/dL, (c) hypertriglyceridemia (triglycerides ≥150 mg/dL), or (d) elevated LDL (≥100 mg/dL) [25] and hypertension (defined as having a blood pressure ≥140/90 mmHg or using any antihypertensive medication). Systolic and diastolic blood pressures were measured twice with at least a 15-minute interval in between and the averages were recorded [26].

### 2.3. Anthropometric Measurements

A body mass index (BMI) ≥25 kg/m^2^ (overweight and obesity) was considered abnormal [27]. Values of waist circumference (≥102 cm for men and ≥88 cm for women) and waist-to-hip ratio (WHR) ≥0.9 for men and ≥0.85 for women were considered indicators of abdominal obesity. Waist circumference was measured around the midpoint between the lowest rib and the iliac crest after exhaling and viewed from the front. Hip circumference was measured at the point of maximum extension of the buttocks when viewed from the side [28]. Abnormal WHR values are consistent with National Cholesterol Education Program Adult Treatment Panel III and World Health Organization definitions [29, 30]. The above measurements were used to find the most reliable predictors of vascular involvement.

### 2.4. CVD and Assessment of Carotid Intima-Media Thickness (IMT)

CVD was categorized as positive if any of the following subphenotypes were present: hypertension, coronary artery disease (CAD), occlusive arterial disease, carotid disease, or thrombosis [20]. In addition, an expert blind to the patients' clinical records performed ultrasound measurements of the IMT from the common carotid artery. High-resolution, 2-dimensional images were obtained using an ultrasound machine (Agilent-Hewlett-Packard, Santa Clara, CA). A 7.5 MHz linear-array transducer with an axial resolution of 0.15 mm and a penetration depth from 1.0 to 5.0 cm was used [31]. The patient rested on the examination table for 15 minutes before the initial carotid ultrasound scan. The measurement was applied to the far wall of the right and left carotid arteries. Following R. Salonen and J. T. Salonen [32], a B-mode screening method was used with electronic calipers within 10 mm proximal to the common carotid bifurcation in a temperature-controlled room (22°C to 24°C) [33]. Ultrasound images were recorded on videotape (Sony MD385). IMT was measured at the site of the greatest thickness and at 2 additional points: 1 cm upstream and 1 cm downstream from this site. The average of these 3 values was computed. Since carotid IMT greater than 0.90 mm is included among the definitions of subclinical organ damage [34], we established this point as having severe subclinical atherosclerosis (AT). The reproducibility of the IMT was evaluated in 12 volunteers, by taking 2 measurements one month apart; obtaining an intraclass correlation of 0.98.

### 2.5. Genotyping

Genomic DNA was extracted from buffy-coat cells using standard methods. All the identified genomic DNA samples were genotyped for* GDF15(MIC1)* rs1058587 at the Oklahoma Medical Research Foundation using the TaqMan allelic discrimination assay (Applied Biosystems). The* GDF15(MIC1)* gene, located at band p13.11 on chromosome 19, has two exons that encode the 308-amino acid* GDF15(MIC1)* polypeptide, consisting of a 29-amino acid signal peptide, a 167-amino acid propeptide, and a 112-amino acid mature protein. Cleavage of the propeptide allows the mature protein to be secreted as a disulfide-linked homodimer. rs1058587 SNP is at codon 202 (**C**AC to** G**AC) and results in a histidine to aspartic acid substitution at position 6 of the mature protein (H6D) (http://www.ncbi.nlm.nih.gov/gene/9518).

### 2.6. Statistical Analysis

Hardy-Weinberg equilibrium was checked in each genotypic marker. Univariate analyses were performed as follows: the categorical variables were analyzed by the frequencies. The quantitative continuous variables were expressed as the mean and standard deviation, as well as the median and range. Bivariate analysis of CVD outcome (subphenotypes and IMT) versus genetic, clinical, and demographic variables was assessed by means of chi square and Kruskal-Wallis test. The mixed cluster methodology proposed by Morineau et al. [35] based on multivariate descriptive methods such as principal components analysis (PCA) and multiple correspondence analysis (MCA) was performed to resume some sets of variables that have strong associations. For example, AOD and duration of disease were analyzed in this setting to derive three groups that resemble the associations of these variables (i.e., time cluster). The same was done for SES and educational level deriving four groups (i.e., sod-cluster) and, for Sjögren's syndrome (SS), EAM, and comorbidity three groups were determined (i.e., clinical cluster). This allows the integration of these sets of variables that are confounded, via the new clusters, in association models like logistic regression and classification and regression trees (CART).

CART were used to find predictive factors for CVD in RA. The CART model was adjusted in the Salford Predictive Modeler software v7 using different splitting rules and cross-validation samples to accurately estimate the relative errors of the classification tree. As the independent factors, the model included the variables that were statistically significant in bivariate analyses and those variables that were biologically plausible, as well as the new clusters derived. Bivariate and multivariate analyses were performed in R 3.0.2 [36].

## 3. Results

Of the 310 patients, 87.4% were women and CVD was reported in 69.5%. The SNP rs1058587 was in Hardy-Weinberg equilibrium (*P* > 0.05). Allelic frequencies corresponding to rs1058587 SNP C and G were 82.7% and 17.5%, respectively. The most frequent genotype was CC (68.4%). The frequencies of RF and ACPA were 68.3.% and 80.4%, respectively. Characteristics of the cohort are illustrated in Table 1. Significant differences concerning* GDF15* polymorphism (rs1058587) were not observed between patients and controls (Table 2).

Data analysed by PCA and MCA were resumed in clusters under clinical, time, and sociodemographic categories. The clinical clusters was gathered into 3 groups: (1) the highest frequency comorbidities (e.g., T2DM, dyslipidemia, osteoporosis, acid peptic disease, kidney disease, depression, periodontal disease, and fibromyalgia), not having polyautoimmunity with SS and moderate frequency of EAM; (2) presence of comorbidities and polyautoimmunity with SS and the highest frequency of EAM; (3) absence of comorbidities, not having polyautoimmunity with SS and lower frequency of EAM. The time clusters was categorized into 3 groups: (1) later AOD and shorter duration of the disease; (2) earlier AOD and longer duration of the disease; (3) earlier AOD and shorter duration of the disease. Finally, the sod-clusters was classified into 4 groups: (1) medium SES and the highest educational level; (2) medium SES and lower educational level; (3) the highest SES and higher educational level; and (4) the lowest SES and higher educational level.

The patients with CVD were significantly older and had a later AOD of RA. Duration of EAM, mean arterial pressure, current smoking, clinical cluster number 1, and time cluster number 1 were all risk factors significantly associated with CVD. Associations between* GDF15 *rs1058587 SNP and CVD (subphenotypes) in RA patients (Table 3) or with IMT were not found.

In Tables 4 and 5 are mentioned the variables associated with the presence of allele C and allele G of the* MIC1 *rs1058587 SNP, respectively. Once again, we did not find associations between this SNP and CVD in those patients.

In the genotype analysis, the homozygotes G/G were significantly older (median 54, IQR 11.7 years), had later AOD (median 43.7, IQR 9.92 years), and had the highest diastolic blood pressure (median 88.33, IQR 8.66 mmHg). On the other hand, the genotype CC was found as protective factor to have positive ANA (OR 0.27; 95% C.I. 0.11–0.85, *P* = 0.019).

### 3.1. CART Predictive Model

**Figure 1:**
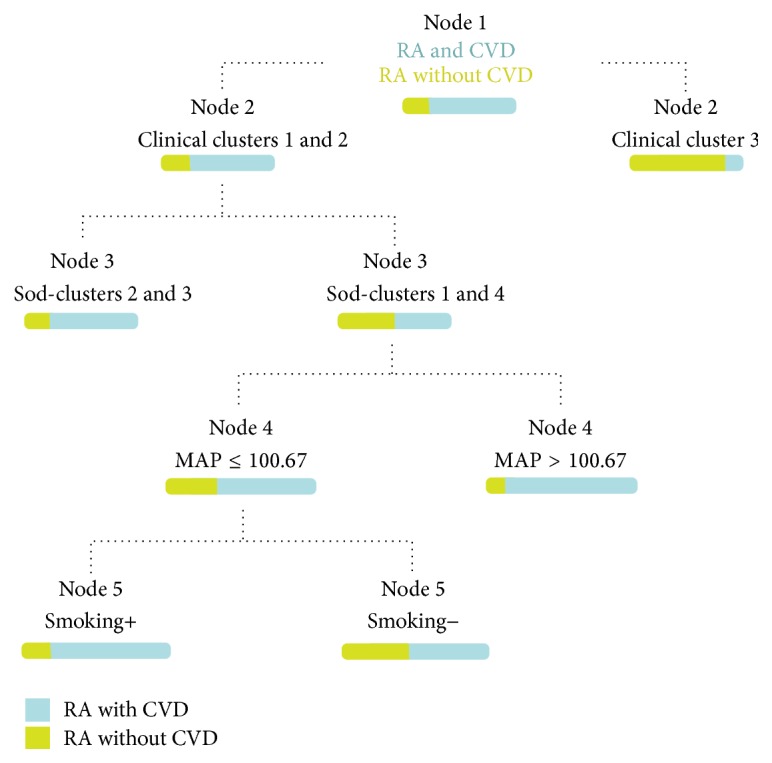
CART predictive model: the main predictive factors significantly associated with the development of CVD in RA are clinical and sociodemographic cluster, mean arterial pressure (MAP), and current smoking, with variable importance scores higher than 0.73.

The CART model displayed a high proportion of patients with RA through clinical clusters 1 and 2 who also develop CVD (nodes 1 and 2). However, there was association between CVD and sod-cluster, mean arterial pressure (MAP), and current smoking; in fact the major association with CVD is in sod-clusters 2 and 3. However, sod-clusters 1 and 4 show a better discrimination for CVD (node 3). Moreover, in the same group of patients, CVD was higher in those with high MAP (i.e., more than 100 mmHg) (node 4). Finally, a greater proportion of CVD was observed in patients in node 4 with low MAP but presenting with current tobacco exposure (node 5) (Figure 1). This model had an AUC of 0.73 (cross-validated AUC), which represents an adequate predictive performance.

## 4. Discussion

Several previous studies of gene polymorphisms in patients with RA who had CVD had demonstrated genetic factors implicated in the development of this comorbidity, such as HLA-DRB1 shared epitope alleles [37, 38]. Moreover, the choice of genes for analysis in those studies was based on a relationship with RA* per se* or with inflammation. Herein, we were unable to replicate the influence of* GDF15(MIC1)* rs1058587 SNP in Colombian patients with RA and CVD as was previously observed in Swedish and Australian patients [11, 14]. Differences in admixture patterns and allele frequencies among populations may account for the observed differences in the influence of genetic factors on disease. Our population is highly admixed with Amerindian, African, and Caucasian ancestries as compared with Australians and Swedish who are mainly Caucasians. The rs1058587 G allele frequency was significantly lower in Colombians than in Australians and Swedish [11, 14].

The general prevalence of CVD in our cohort was 69.5%. Some associations between RA patients with CVD were found. Variables that were significantly associated include smoking, higher MAP, longer duration of disease with EAM, clinical cluster number 1, and time cluster number 1. It is important to highlight that these associations encompass only clinical variables that include modifiable traditional risk factors for CVD like smoking and nontraditional risk factors including duration of disease and EAM [5].

*GDF15(MIC1)* rs1058587 C allele was nevertheless associated with minor frequency of smoking and obesity, lower BMI, and lower measurements of diastolic blood pressure (Table 4), whereas G allele was associated with earlier AOD, clinical cluster number 1, and positive ANA and it was inversely associated with SS and time cluster number 1. Although modest, these findings contrast with reports showing a specific interaction between the G allele and smoking, with an increase of risk for stroke [11].

At the genotype level a statistically significant association was found between G/G genotype and high diastolic blood pressure, while C/C genotype was inversely associated with positive ANA, suggesting a protective role for polyautoimmunity. We were not able to find other studies reporting associations between the rs1058587 homozygote forms and CVD or RA* per se*. As it is shown, these associations do not confirm a direct relationship between *MIC1* and CVD.

Many reasons exist why no significant association was elicited within the cohort and the gen evaluated with the presence of CVD. One of them is the sample size calculation since it was a nonprobability sample and all RA patients from this region were not included in the study. Besides, the heterogeneity of the cohort may prevent us from finding a direct association. A group of patients studied constitute a closed subpopulation into the admixed Colombian community (i.e., Medellín) and another group represents a major variation in ancestry. Otherwise, previous publications addressing the influence of* GDF15(MIC1)* in CVD were made in general population, not in RA patients. Therefore, the interaction among autoimmunity, admixture population, and genetic, epigenetic, and environmental factors could influence the behavior of* GDF15(MIC1)* in our study.

## 5. Limitations of the Study

The aim of this study was to determine the influence of rs1058587 SNP within* GDF15(MIC1)* gene on the risk of CVD in a Colombian RA population and we are aware of our study limitations. First of all, selection bias could be present in our analysis as not all patients from this region with RA were systematically included. Secondly, although the study sample size is not negligible, it would have been more valuable to have had an appropriate follow-up to establish valid associations between CVD,* GDF15(MIC1),* and RA. This, in turn, could have improved both internal and external validities. Finally, the cross-sectional nature of the study does not allow us to infer causality. Although we were able to evaluate some associations between* GDF15(MIC1) *and risk factors for CVD, it would have been more valuable to have a follow-up, as it is possible in cohort multicenter studies, to validate and establish more associations between this gene, CVD, and RA.

## 6. Conclusions

Contrary to prior reports, we could not find an association between the SNP rs1058587 of the gene* GDF15* and the development of CVD (measured through subphenotypes or IMT) in the population studied. There was an association between this SNP and traditional and environmental factors for CVD (i.e., age, obesity, and smoking) and some nontraditional risk factors (i.e., ANA and SS). However, we had not found direct association between the polymorphism and CVD. In fact, MAP, EAM, smoking, duration of the disease, and AOD are highlighting the pivotal role of these factors in our population. CVD in RA patients should be approached following the 5P rule: (1) predicting patients at risk of developing CVD; (2) preventing CVD in susceptible patients; (3) personalizing the approach of CVD based on the premise that susceptibility and severity of the disease are unique in each patient; (4) allowing the patient to participate in the decisions made in order to prevent and treat CVD; and (5) incorporating all data and policies according to the population, since the characteristics and natural history of diseases are population-specific [39].

## Figures and Tables

**Table 1:** Demographic and clinical characteristics of 310 patients with RA.

Variable	Median (range)
Age (y)	37 (20–54)
Age at onset (y)	50 (26–67)
Duration of the disease (y)	12 (1–20)
BMI	29 (19–37)
EAM duration (m; media ± SD)	11.7 ± 3.3

Genetics	*n*/*N* (%)

rs1058587 CC	212/310 (68.4)
rs1058587 CG	88/310 (28.4)
rs1058587 GG	10/310 (3.22)
Allele C	513/620 (82.7)
Allele G	109/620 (17.5)

Sociodemographic	*n*/*N* (%)

Female	271/310 (87.4)
Civil status: married	174/282 (61.7)
Occupation: household duties	109/287 (38)
Socioeconomic status: medium	195/278 (70.1)
High educational level	169/212 (79.7)
Current smoking	49/272 (18)

RA-related	*n*/*N* (%)

Polyautoimmunity: SS	16/310 (5.1)
Familial autoimmunity	47/272 (17.3)
EAM^&^	117/303 (38.6)
Comorbidity^#^	225/305 (73.7)
Rheumatoid factor (+)	162/237 (68.3)
ACPA (+)	107/133 (80.4)
ANA (+)	31/75 (41.3)
Erosive (+)	99/110 (90)
Methotrexate	248/273 (90.8)
DMARDs, ever^¥^	83/117 (71)
Antimalarials, ever^*£*^	158/256 (61.7)
Steroids, ever^æ^	250/269 (92.9)
Biological therapy, ever^€^	37/256 (14.4)

Cardiovascular	*n*/*N* (%)

Cardiovascular disease^*₳*^	130/187 (69.5)
cIMT abnormal	66/118 (55.9)
Familial cardiovascular disease	19/245 (7.7)
BMI: obesity	18/118 (15.2)
BMI: overweight	42/118 (35.6)
Physical activity	20/121 (16.5)
Abnormal waist-hip ratio	90/117 (76.9)

ACPA: anti-citrullinated protein antibodies; ANA: anti-nuclear antibodies; BMI: body mass index; cIMT: carotid intima-media thickness; DMARDs: disease modifying antirheumatic drugs; EAMs: extra-articular manifestations; HLA: human leukocyte antigen; m: months; *MIC1*: macrophage inhibitory cytokine-1; RA: rheumatoid arthritis; SD: standard deviation; SS: Sjögren's syndrome; y: years.

^&^EAM is defined as the presence of at least one of the following: nodules, skin ulcerations, episcleritis, scleritis, vasculitis, neuropathy, pleural effusion, pulmonary nodules, or pulmonary hypertension.

^#^It is defined as the presence of at least one of the following: type 2 diabetes mellitus, dyslipidemia, kidney disease, anemia, osteoporosis, depression, fibromyalgia, acid peptic disease, epilepsy, or infections (e.g., hepatitises A, B, and C, tuberculosis, or malaria).

^¥^If patient had used at least one of the following: sulfasalazine, D-penicillamine, gold salts, and leflunomide.

^*£*^If patient had used at least one of the following: chloroquine and hydroxychloroquine.

^æ^If patient had used at least one of the following: prednisolone, methylprednisolone, and deflazacort.

^€^If patient had used at least one of the following: etanercept, infliximab, adalimumab, abatacept, tocilizumab, and rituximab.

^*₳*^It was categorized as positive if any of the following variables were present: hypertension, coronary artery disease, occlusive arterial disease, carotid disease, or thrombosis.

**Table 2:** *GDF15* polymorphism (rs1058587) in Colombian patients with RA.

Genotype	RA *N* = 310 (%)	CTR^*^ *N* = 228 (%)
CC	212 (68.4)	151 (66.2)
CG	88 (28.4)	70 (30.7)
GG	10 (3.2)	7 (3.1)
Allele	2*N* = 620	2*N* = 456
C	512 (82.6)	372 (81.6)
G	108 (17.4)	84 (18.4)

^∗^Significant differences were not observed.

**Table 3:** Characteristics associated with CVD in RA patients.

Variable	CVD	Non-CVD		*P* value
	130/187 (69.5)	57/187 (30.5)		
	Median (IQR)	Median (IQR)		
Duration of the disease (y)	14.6 (9.038)	9 (5.565)		<0.001
EAM (m)	17.115 (37.6)	4.962 (9.559)		0.028
Mean arterial pressure	99.709 (12.992)	95.491 (11.605)		0.0361

Variable	ECV	Non-ECV	OR (95% CI)	*P*
	*n*/*N* (%)	*n*/*N* (%)		

Clinical cluster 1^&^	112/130 (86.1)	32/57 (56.1)	33.93 (8.6–259.7)	<0.001
Time cluster 1^#^	62/130 (47.6)	24/57 (42.1)	1.99 (1.07–4.16)	0.0274
Current smoking	29/130 (22.3)	4/57 (7.4)	2.54 (1.01–8.45)	0.0312
*GDF15* rs1058587 CC	89/130 (68.4)	36/57 (63.1)	—	NS
*GDF15* rs1058587 CG	35/130 (26.9)	21/57 (36.8)	—	NS
*GDF15* rs1058587 GG	6/130 (4.6)	0/57 (0)	—	NS
Allele C	213/260 (81.9)	93/114 (81.5)	—	NS
Allele G	41/260 (15.7)	21/114 (18.4)	—	NS

CVD: cardiovascular disease; EAMs: extra-articular manifestations; *GDF15*: growth differentiation factor 15; IQR: interquartile range; y: years; m: months; NS: not significant; RA: rheumatoid arthritis.

^&^Clinical cluster 1 corresponds to patients with the highest frequency of comorbidity, with moderate frequency of EAM, and without Sjögren's syndrome.

^#^Time cluster 1 corresponds to patients with later age at onset and shorter duration of the disease.

**Table 4:** Characteristics associated with allele C of the *GDF15* rs1058587 SNP in RA patients.

Variable	Allele C (CC/CG)	Allele G (GG)		*P* value
	300/310 (96.7)	10/310 (3.22)		
	Median (IQR)	Median (IQR)		
Diastolic blood pressure	80.662 (10.437)	88.333 (8.66)		0.0286
Body mass index	25.326 (4.691)	29.322 (4.177)		0.0457

Variable	Allele C	Allele G	OR (95% CI)	*P* value
	*n*/*N* (%)	*n*/*N* (%)		

Current smoking	45/300 (15)	4/10 (40)	0.14 (0.02–0.80)	0.016
Obesity	15/300 (5)	3/10 (30)	0.09 (0.009–0.97)	0.0175
CVD	124/300 (41.3)	6/10 (60)		NS

CVD: cardiovascular disease; IQR: interquartile range; RA: rheumatoid arthritis.

**Table 5:** Characteristics associated with allele G of the *GDF15* rs1058587 SNP in RA patients.

Variable	Allele G (GG/CG)	Allele C (CC)		*P* value
	98/310 (31.6)	212/310 (68.3)		
	Median (IQR)	Median (IQR)		
Age (y)	49.398 (12.46)	53.986 (11.976)		0.001
Age at onset (y)	37.469 (11.513)	41.344 (12.59)		0.008

Variable	Allele G	Allele C	OR (95% CI)	*P* value
	*n*/*N* (%)	*n*/*N* (%)		

Clinical cluster 1^&^	72/98 (73.46)	129/212 (60.84)	2.88 (1.16–9.32)	0,0145
ANA (+)	15/98 (15.30)	16/212 (7.54)	2.72 (1.15–8.21)	0,0203
Sjögren's syndrome	4/98 (4.08)	26/212 (12.26)	0.28 (0.11–0.91)	0,0209
Time cluster 1^#^	32/98 (32.63)	98/212 (46.22)	0.52 (0.31–0.92)	0,0228
CVD	41/98 (41.88)	89/212 (41.98)		NS

ANA: anti-nuclear antibodies; CVD: cardiovascular disease; IQR: interquartile range; RA: rheumatoid arthritis.

^&^Clinical cluster 1 corresponds to patients with the highest frequency of comorbidity, with moderate frequency of EAM, and without Sjögren's syndrome.

^#^Time cluster 1 corresponds to patients with later age at onset and shorter duration of the disease.
